# Association of Serum Zinc and Inflammatory Markers with the Severity of COVID-19 Infection in Adult Patients

**DOI:** 10.3390/nu15020340

**Published:** 2023-01-10

**Authors:** Abdulaziz Saad Almasaud, Jamal Chalabi, Abdulmajid Al Arfaj, Ali Al Qarni, Ammar Alkroud, Zuheb Nagoor, Sana Akhtar, Jahangir Iqbal

**Affiliations:** King Abdulaziz Hospital, King Abdullah International Medical Research Center-Eastern Region, King Saud Bin Abdulaziz University for Health Sciences, Ministry of National Guard-Health Affairs, Al Ahsa 31982, Saudi Arabia

**Keywords:** COVID-19, SARS-CoV-2, zinc, inflammatory markers, viral infection

## Abstract

COVID-19 infection can spread in the host body without any adequate immune response. Zinc is an essential trace element with strong immunoregulatory and antiviral properties and its deficiency might lead to inflammation and oxidative stress. The aim of the current study was to determine the association of serum zinc and inflammatory markers with the severity of COVID-19 infection. This was a prospective observational study in which 123 COVID-19-positive adult patients and 48 controls were recruited. The initial comparative analysis was conducted between COVID-19 patients and controls. COVID-19-positive patients were further divided into three different groups (mild, moderate, and severe) based on the severity of COVID-19 infection. COVID-19 patients showed significantly lower serum zinc levels (8.8 ± 2.3 µmol/L) compared to healthy controls (11.9 ± 1.8 µmol/L). There was a negative correlation between serum zinc levels and the severity of COVID-19 infection (r = −0.584, *p* < 0.0001) and this effect was independent of age (r = −0.361, *p* < 0.0001). Furthermore, inflammatory markers showed a positive correlation with the severity of COVID-19 infection and a negative correlation with the levels of serum zinc. The study demonstrated an association between COVID-19 infection with low serum zinc levels and elevated inflammatory markers. Further studies to assess the significance of this observation are needed, which may justify zinc supplementation to mitigate the severity of COVID-19 infection.

## 1. Introduction

The novel coronavirus disease of 2019 (COVID-19) caused by severe acute respiratory syndrome coronavirus (SARS-CoV2) in humans, represents the largest current health challenge for the society. Viral infections lead to the upregulation of immune response to clear infections and, therefore, decreased immunity is a significant risk factor for viral infections. COVID-19, in addition to upregulating pro-inflammatory cytokines such as interleukin 1 (IL-1), IL-6, and tumor necrosis factor (TNF-α) also leads to higher concentrations of the anti-inflammatory cytokine IL-10 [1]. Patients with COVID-19 likely have evidence of oxidative stress and a concomitant deficiency of antioxidants [2]. A proper nutritional status and good diet are important factors for an optimal immune response to prevent viral infection. Zinc is one of the major factors that control normal development, differentiation, and function of immune cells involved in cell-mediated immunity against any infectious agent by modulating cytokine production [3]. Zinc is an essential trace element with potent antiviral and immunoregulatory properties. Zinc-deficiency not only hampers our immune system, but it can also be associated with an excessive release of pro-inflammatory cytokines. It possesses a variety of direct and indirect antiviral properties and protects cells from the adverse effect of reactive oxygen species (ROS) produced during inflammation [4]. Zinc is also known to be important for immune function and has a role in antibody production. A deficiency of zinc affects the function of the innate immune system and is associated with reduced antibody production and decreased cytokine production with a concomitant increase in pro-inflammatory cytokines [5].

Autophagy plays an important protective role as a host defense mechanism in certain pathological conditions including viral infection [6,7,8]. Under basal conditions, depletion of zinc was shown to cause suppression of autophagy in a cell culture model and its supplementation stimulated autophagy in the same cells [9]. Zinc has also been implicated in coronavirus biology. Increased intracellular concentrations of zinc were shown to inhibit viral replication in an in vitro cell culture model of SARS-CoV1 and other viruses [10,11,12,13]. Furthermore, zinc treatment was shown to augment the production of interferon (IFNα) by leukocytes [14] and potentiate its antiviral activity in rhinovirus-infected cells [15]. These important findings demonstrated intracellular zinc as a potential inhibitor of the replicative cycle of these viruses and suggest that zinc may be considered as the particular antiviral agent in COVID-19 treatment [16]. A negative association between zinc deficiency and COVID-19 severity was found in one observational study [17].

Adequate zinc levels are essential to provide an enhanced immune response, anti-inflammatory, and other protective effects against COVID-19 infection. Zinc acts as an immunomodulatory and plays an important role in the process of immune development. Zinc deficiency has been associated with reduced macrophage activation and poor development of lymphoid tissue [18,19]. Studies have shown that SARS-CoV-2 viral spike protein enters the cells after interacting with angiotensin-converting enzyme 2 (ACE2) which is a zinc-dependent hydrolase [20]. Inflammatory markers may be associated with disease severity and elevation of C-reactive protein (CRP), procalcitonin (PCT), lactate dehydrogenase (LDH), and ferritin has been shown in patients with severe, and not mild, COVID-19 infection [21]. This elevation may be associated with cytokine storm, septic shock, and organ failure and may be a good predictor of the severity of the COVID-19 infection. There is not much published data about the correlation between serum zinc levels and COVID-19 infection severity. Hence, the current study aimed to identify the association between serum zinc and inflammatory markers with the severity of COVID-19 infection in adult patients.

## 2. Materials and Methods

### 2.1. Materials

Alinity c CRP kit (catalog #07P5621), Alinity i PCT kit (catalog #01R1821), Alinity c LDH kit (catalog #07P7420), and Alinity i Ferritin kit (catalog #07P6520) were purchased from Abbot Labs (Des Plaines, IL, USA). Standard solution of zinc was prepared from a 10 µg/mL multi-element solution from Agilent Technologies (Santa Clara, CA, USA). Germanium internal standard 10 µg/mL internal standard mix was from Agilent Technologies (Santa Clara, CA, USA). Two lyophilized serum controls (UTAK, Trace Element Serum Control Level 1-66816, and Level 2-66815, Valencia, CA, USA) were used as reference materials. Higher purity trace metal grade nitric acid (Fisher Scientific, Loughborough, UK) was used for acidification of zinc determination solution. Ultrapure water used for this study was obtained using a Milli–Q system (Millipore, Bedford, MA, USA). All other chemicals and solvents were obtained from Fisher Scientific through its local distributor in the Kingdom of Saudi Arabia.

### 2.2. Study Design

This was an observational prospective study that evaluated 123 patients diagnosed with COVID-19 presenting to either the intensive care unit (ICU), wards, or Health Clinic of King Abdulaziz Hospital, Ministry of National Guard Health Affairs in Al Ahsa, Saudi Arabia. The data were collected from 25 June 2020 to 3 March 2022. The study was approved by the Institutional Review Board ethics committee of the Ministry of National Guard Health Affairs (Research Protocol #RA20/027/A).

### 2.3. Patients and Data Collection

Patients were recruited and characterized into four groups based on the criteria of the Saudi Ministry of Health (MOH) criteria:

1. Severe cases: COVID-19-positive patients who required admission to ICU (usually in respiratory distress or require>50% O_2_).
2. Moderate cases: COVID-19-positive patients who required admission to the ward (usually requires <50% O_2_) and did not require ICU admission during their illness.
3. Mild cases: COVID-19-positive patients who did not require hospital admission but home isolation (no O_2_ requirements).
4. Control cases: Individuals who came to respiratory clinic with symptoms suggestive of COVID-19 infection and tested negative.

Inclusion criteria for recruitment were:

1. Adult patients (18 years or older) who were symptomatic and tested positive for COVID-19 at King Abdulaziz Hospital whether they required admission to ICU/ward or just home isolation.
2. Individuals who came to respiratory clinic with symptoms suggestive of COVID-19 infection and tested negative.

The exclusion criteria included:

1. Documented other infections apart from COVID-19.
2. Any participant who was prescribed zinc supplement prior to blood sampling.
3. Receiving immune-suppressant or immune-modulatory medications within 90 days prior to recruitment.

Symptoms and signs suggestive of COVID-19 infection were coughs, shortness of breath or difficulty breathing, subjective measured fever (documented temperature >38 °C), chills or shivering, fatigue, muscle or body aches, diarrhea, nausea, vomiting, headache, sore throat, runny nose, and/or loss of smell or taste.

Informed consent was taken from clinic patients to withdraw blood and to give their demographic data and medical history. No consents were required from admitted patients since blood work was part of the routine labs taken for all admitted COVID-19 patients.

### 2.4. Serum Zinc, Biochemical and Hematological Assessment

Blood (5 mL) was collected in trace metal-free vacuum tubes from each subject at the time of either admission or recruitment for zinc and biochemical determination. Blood samples were centrifuged within 30 min of collection and sera were stored at −20 °C for further evaluation. An amount of 5 mL of blood was also collected from each subject in evacuated tubes containing EDTA for hematological assessment. Serum zinc levels were measured using inductively coupled plasma—mass spectrometry (Agilent 7700 series ICP-MS, Agilent Technologies Inc., Santa Clara, CA, USA) using argon gas to generate the plasma [22]. The instrument features octopole reaction system—collision/reaction cell which is pressurized with helium gas to remove matrix-based inferences that inhibit zinc analysis. Diluted nitric acid was used as a carrier solution for introducing the sample to ICP-MS. Levels of PCT and ferritin were determined using commercially available chemiluminescent microplate immunoassay kit and levels of LDH were determined using commercially available kinetic colorimetric method kit from Abbott Diagnostic and analyzed on Alinity ci-Chemistry Analyzer according to the recommendation of the IFCC. Levels of CRP were determined using commercially available kit from Abbott and analyzed by means of an immunoturbidimetric method that absorb the agglutination at 572 nm on Alinity ci-Chemistry Analyzer, Abbott Diagnostic. The determination of white blood in human whole blood was performed by Alinity hg-Blood Analyzer, Abbott Diagnostic. All the serum measurements were performed in duplicate.

### 2.5. Statistical Analysis

The statistical analysis was performed using GraphPad Prism (version 5.0; GraphPad, San Diego, CA, USA) and IBM SPSS Statistics (version 20; IBM, New York, NY, USA) software. Numerical data with normal distribution are expressed as mean ± standard deviation (mean ± SD), and comparisons among the groups were performed using the one-way analysis of variance (ANOVA). Nominal data were analyzed by chi-square test. Correlation was analyzed by Pearson correlation analysis. The statistical analysis of differences between two groups was performed using two-tailed Student’s *t*-test with a confidence level of 95%. A *p* value of <0.05 was considered statistically significant.

## 3. Results

### 3.1. Basic Characteristics and Comparisons between COVID-19 Patients and Controls

In this observational prospective study, a total of 123 adult COVID-19 patients and 48 controls (≥18 years old) were enrolled. The comparative analysis of COVID-19 patients and controls showed a median age of 59.0 years (range 19–108 years) versus 33.0 years (range 21–56 years). COVID-19 patients had significantly lower zinc levels of 8.8 ± 2.3 µmol/L in comparison to the controls at 11.9 ± 1.8 µmol/L (Table 1). We found a significant increase in other biochemical and hematological parameters for COVID-19 patients including WBC, CRP, PCT, LDH, and ferritin (Table 1).

**Figure 1:**
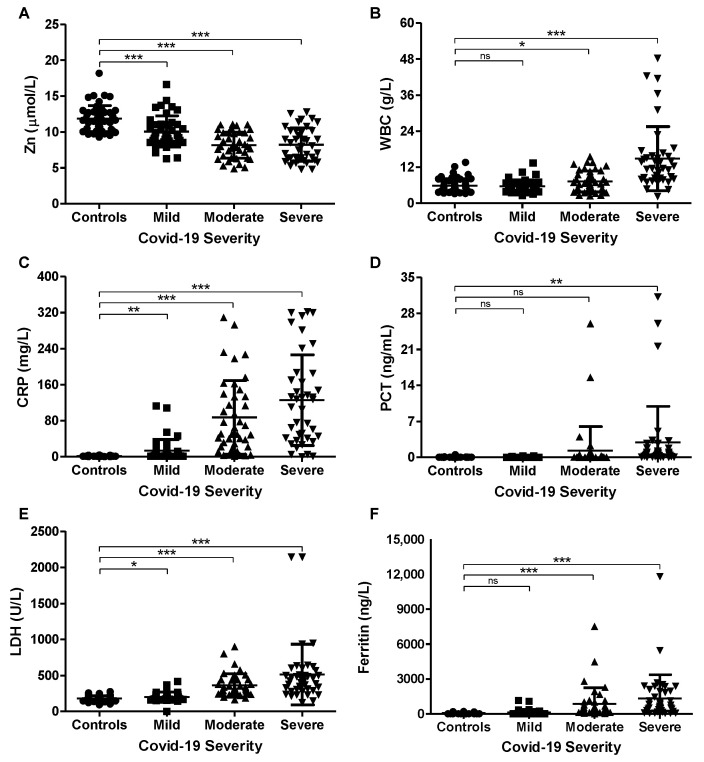
Serum zinc and inflammatory marker levels in controls and COVID-19 patients. Blood from controls (*n* = 48), mild (*n* = 42), moderate (*n* = 41), and severe (*n* = 40) COVID-19 subjects was used to assess zinc (**A**), WBC (**B**), CRP (**C**), PCT (**D**), LDH (**E**), and ferritin (**F**) levels. Values are plotted as replicates (mean ± SD). * *p* < 0.05, ** *p* < 0.01, and *** *p* < 0.001. ns, not significant.

Based on the severity of the 123 patients with COVID-19 infection, subjects were classified as mild 42 patients (34%), moderate 41 patients (33.5%), and severe 40 (32.5%). The descriptive statistics of the study population are presented in Table 2. The distribution of gender did not differ significantly between mild, moderate, and severe COVID-19 groups (*p* = 0.839). However, patients with moderate or severe COVID-19 were significantly older compared to patients in the mild group, and the age in years ± SD was (65.6 ± 13.5), (68.6 ± 16.7), and (35.3 ± 9.9), respectively. Furthermore, only 1 death (2.4%) out of the total of 41 patients in the moderate group occurred in contrast to 25 deaths (62.5%) that occurred in the severe group. Serum zinc levels were significantly lower in the moderate and severe groups compared to the mild group (Figure 1A and Table 2). On the other hand, serum levels of WBC (Figure 1B), CRP (Figure 1C), PCT (Figure 1D), LDH (Figure 1E), and ferritin (Figure 1F) showed an increasing trend with the increase in the severity of the infection (Table 2).

Next, we analyzed the COVID-19-positive patients who were admitted to the hospital (moderate and severe) based on their hospital outcomes as death or discharge (Table 3). Overall, there were significant statistical differences in age as patients who died were older, presentation with a higher level of serum levels of WBC, CRP, PCT, and LDH. However, there was no statistically significant difference in the levels of serum zinc and ferritin between hospital discharge and death patients.

### 3.2. Association of Serum Zinc with COVID-19 Severity and Inflammatory Markers 

**Figure 2:**
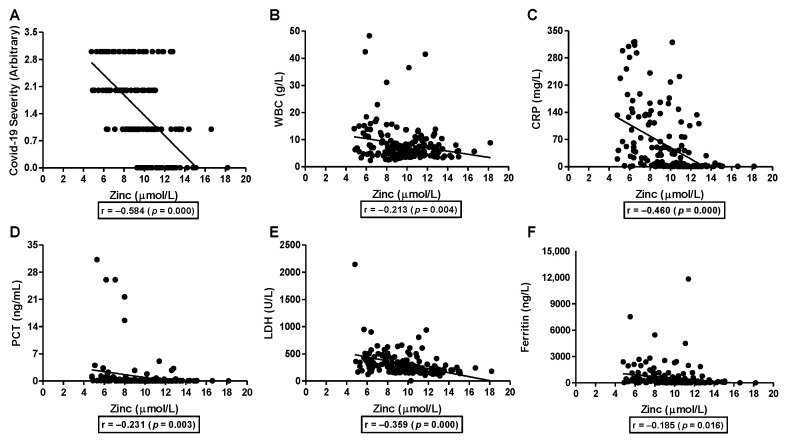
Correlation between serum zinc and inflammatory markers in the COVID-19 patients. Serum zinc value from all the subjects (control and COVID-19 patients) was used to study the association with COVID-19 severity (**A**), WBC (**B**), CRP (**C**), PCT (**D**), LDH (**E**), and ferritin (**F**). Correlation was analyzed by Pearson correlation analysis.

Serum zinc levels showed a moderately significant negative correlation with the severity of COVID-19 infection (r = −0.584, Figure 2A). There was also a significant negative very weak to moderate association between serum zinc and inflammatory markers WBC (r = −0.213, Figure 2B), CRP (r = −0.460, Figure 2C), PCT (r = −0.231, Figure 2D), LDH (r = −0.359, Figure 2E), and ferritin (r = −0.185, Figure 2F).

**Figure 3:**
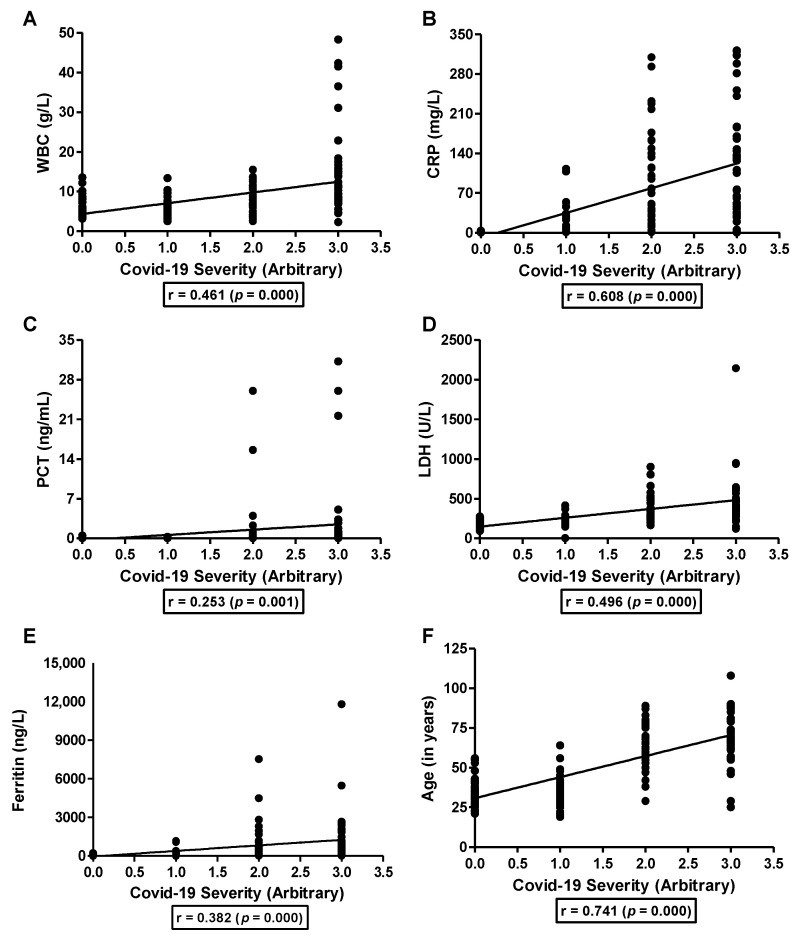
Association of inflammatory markers with the severity of COVID-19 infection. Severity of COVID-19 infection was used to study the association with WBC (**A**), CRP (**B**), PCT (**C**), LDH (**D**), ferritin (**E**), and age (**F**). Correlation was analyzed by Pearson correlation analysis.

Next, we looked at the association of inflammatory markers with COVID-19 severity. WBC (r = 0.461, Figure 3A), CRP (r = 0.608, Figure 3B), PCT (r = 0.253, Figure 3C), LDH (r = 0.496, Figure 3D), and ferritin (r = 0.382, Figure 3E) showed a moderate positive correlation with the severity of COVID-19 infection. The severity of COVID-19 infection showed a strong direct association with age (r = 0.741, Figure 3F) suggesting that older people were more likely to develop severe COVID-19 infection than younger adults. Furthermore, we observed that serum zinc levels were negatively correlated to the age of the patients (r = −0.506, *p* = 0.000). Therefore, we looked at the effect of age as a confounding factor on the correlation between serum biochemical markers and the severity of COVID-19 infection. The association of serum zinc levels with inflammatory markers was lost after adjustment for age, except for CRP where weak statistically significant negative association persisted with r = −0.238 and *p* = 0.002. Furthermore, the weak association of serum zinc with the severity of COVID-19 infection remained significant after adjusting for age (r = −0.361, *p* = 0.000). Similarly, except for PCT (r = 0.112, *p* = 0.147) and LDH (r = 0.116, *p* = 0.130), the positive association of WBC (r = 0.281, *p* = 0.000), CRP (r = 0.442, *p* = 0.000) and ferritin (r = 0.181, *p* = 0.018) with COVID-19 severity remained significant after adjusting for age albeit week. We also found a positive moderate correlation between the severity of COVID-19 and mortality (r = 0.566, *p* = 0.000) which remained significant after adjustment for age (r = 0.330, *p* = 0.000).

### 3.3. Association of Serum Zinc with Number of Comorbidities and APACHE II Score

**Figure 4:**
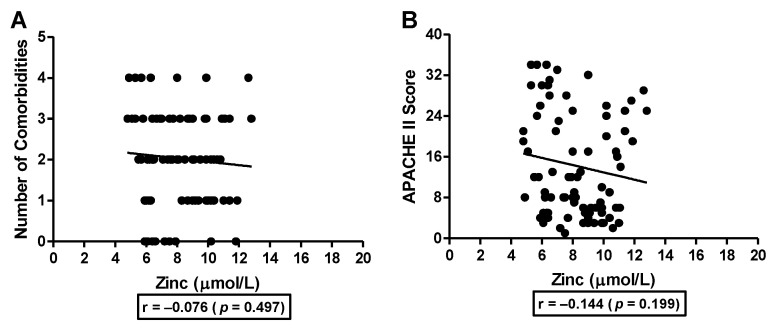
Association of number of comorbidities and APACHE II scores with serum zinc levels in hospitalized COVID-19 subjects. Number of comorbidities (**A**) and APACHE II score (**B**) were used to study the association with serum zinc levels in moderate and severe COVID-19 subjects admitted to either the hospital wards or ICU, respectively. Correlation was analyzed by Pearson correlation analysis.

Next, we looked at the association of serum zinc levels with the number of comorbidities and APACHE II (Acute Physiology And Chronic Health Evaluation II) scores in moderate and severe COVID-19 patients admitted to either the hospital wards or ICU, respectively. APACHE II score is a general measure of disease severity based on current physiologic measurements, age, and previous health conditions. Based on the present data, we did not find any significant association of serum zinc levels with the number of comorbidities (r = −0.076, Figure 4A) and the APACHE 11 score (r = −0.144, Figure 4B) in the hospital-admitted COVID-19 subjects. These data suggest that an increased number of comorbidities or higher APACHE II score does not influence the serum zinc levels in the COVID-19 subjects.

## 4. Discussion

The ongoing COVID-19 infection has attracted researchers to explore predictors of disease severity that can aid in combating the SARS-CoV-2 virus. The aim of this study was to determine the association of serum zinc and inflammatory markers with the severity of COVID-19 infection in adult patients. Our study showed that older patients are more likely to develop severe COVID-19 symptoms compared to younger adults which was in line with other published study [23]. Analysis of biochemical and hematological parameters such as WBC, CRP, LDH, and ferritin levels in serum showed a significant increase in COVID-19 patients compared to controls which was consistent with a study reported by Asghar et al. [24]. We observed a 10-fold increase in the levels of serum ferritin in severe COVID-19 subjects compared to mild patients. This was consistent with a meta-analysis study completed by Kaushal et al. [25] showing severe COVID-19 patients had higher serum ferritin compared with mild patients. Furthermore, we found that 62.5% of the ICU-admitted patients died due to the severity of COVID-19 infection and these dead patients showed significantly higher levels of WBC, CRP, PCT, and LDH compared to hospital-discharged patients. There was a positive correlation between these inflammatory markers with the severity of COVID-19 infection which was consistent with a published study showing significantly increased levels of inflammatory markers in patients experiencing a severe course of disease [24]. 

Serum zinc levels were significantly lower in patients with moderate and severe COVID-19 infection compared to patients with mild COVID-19 infection or controls. These data were consistent with studies showing that severe COVID-19 patients had significantly lower serum zinc levels compared to control subjects [17,26]. Both these studies, however, did not use any control subjects to correlate their data. Serum zinc levels were negatively correlated with the level of inflammatory markers, but this correlation was affected by the confounding factor of age. There was a negative association between serum zinc levels and the severity of COVID-19 infection, which remained significant after adjusting for age. Jahromi et al. [17] observed that the association of serum zinc levels with COVID-19 severity was lost following the adjustment of confounding factors. The authors of this study suggest that very low serum zinc levels (below the normal range) in the studied subjects could be the reason for this effect. Interestingly, we did not see any statistically significant difference in the levels of serum zinc between hospital-discharged and dead patients who were admitted to the hospital after the COVID-19 infection. However, patients in the death event group showed significantly higher levels of inflammatory markers compared to the hospital discharge group. 

One of the limitations of our study is that we did not collect the data regarding other comorbidities such as diabetes, obesity, hypertension, heart failure, etc., in controls and mild COVID-19 subjects and, therefore, were unable to adjust for these parameters during our association analysis. However, we used the comorbidities data collected from the moderate and severe COVID-19 subjects hospitalized in either the wards or ICU, respectively, and correlated it with the serum zinc levels. Interestingly, an increased number of comorbidities was not associated with the serum zinc levels in these subjects suggesting that serum zinc levels are not affected by the number of comorbidities. Furthermore, we did not see any association between the APACHE II score which is a general measure of disease severity based on current physiologic measurements, age, and previous health conditions with the serum zinc levels. These data again reiterate that age and previous health conditions do not affect serum zinc levels in COVID-19 subjects. Another limitation of the study is that we did not define the primary outcome of the study before the data analysis and did not correct for multiple testing. A number of review articles have discussed the relationship between zinc deficiency and COVID-19 and suggested that zinc deficiency may be linked to the severity of COVID-19 infection [27,28,29]. Based on the observation that serum zinc levels are low in hospitalized patients, it may be postulated that zinc is mobilized from the plasma into cells by the influence of a cytokine stress [30]. This bio-redistribution of zinc is essential for protein synthesis, neutralization of reactive oxygen species, and maintaining tissue integrity of human lung epithelium under conditions of inflammatory stress during viral infection to prevent cell apoptosis and mechanical dysfunction [30,31].

Studies have shown that a decrease in the levels of zinc enhances the interaction of ACE2 with SARS-CoV-2 spike protein and an increase in the levels of zinc inhibits this interaction [32,33]. These in vitro studies suggest that zinc may have an important role in COVID-19 infection and its deficiency may not be just a mere association. Further experimental and clinical studies are required to investigate the potential role of zinc deficiency and elevated inflammatory markers in COVID-19 susceptibility. Moreover, further studies are needed to explore whether zinc supplementation can improve COVID-19 infection and if so, unravel the cellular and molecular mechanisms involved in alleviating the severity of COVID-19 symptoms by this mineral.

## 5. Conclusions

In conclusion, this study showed that COVID-19 patients had reduced levels of serum zinc compared to healthy adults. In addition, our study showed a strong positive correlation between inflammatory markers (WBC, CRP, PCT, LDH, and ferritin) with the severity of COVID-19 infection as emphasized in previous studies. Furthermore, patients with moderate and severe COVID-19 infection had lower serum zinc levels. To conclusively show that subjects with lower serum zinc levels will develop severe COVID-19 infection, a well-designed prospective study needs to be performed, which is beyond the scope of the current study. This cause–effect relationship could also be explained by reverse causality. It is possible that low serum zinc levels in COVID-19 subjects may be a result of the higher severity of the infection (the more severe case the lower the zinc level). Although we did not find a significant association between serum zinc levels and mortality in infected patients with COVID-19, our study raises the question of whether zinc supplementation to COVID-19 patients who have low serum zinc or are admitted with moderate to severe infection may improve the clinical outcomes. A study by Gordon and Hardigan [34] found that supplementation of oral zinc was associated with a reduced risk of developing symptomatic COVID-19. Several studies are warranted to look into this idea and, therefore, this could be a good opportunity for further investigation.

## Figures and Tables

**Table 1:** Demographic and laboratory parameters of control subjects and combined COVID-19-positive patients.

Parameters	Controls (48)	COVID-19 Positive (123)	*p*
Age (years)	33.5 ± 8.3	56.2 ± 20.3	0.000
Zinc (µmol/L)	11.9 ± 1.8	8.8 ± 2.3	0.000
WBC (g/L)	5.83 ± 2.36	9.19 ± 7.60	0.003
CRP (mg/L)	1.6 ± 0.9	74.6 ± 88.7	0.000
PCT (ng/mL)	0.03 ± 0.07	1.37 ± 4.90	0.060
LDH (U/L)	180.4 ± 37.6	356.5 ± 286.5	0.000
Ferritin (ng/L)	61.0 ± 61.2	758.7 ± 1483.0	0.001

Data are presented as mean ± SD. The statistical analysis of differences between two groups was performed using two-tailed Student’s *t*-test with a confidence level of 95%. A *p* value of <0.05 was considered statistically significant.

**Table 2:** Demographic and laboratory parameters of control group and COVID-19 groups according to the severity of infection.

Parameters	Controls	Mild	Moderate	Severe	*p*
Age (years) (*n*)	33.5 ± 8.3 (48)	35.3 ± 9.9 (42)	65.6 ± 13.5 (41)	68.6 ± 16.7 (40)	0.000
Sex (M/F)	23/25	17/25	19/22	20/20	0.839
Death (%)	0 (0%)	0 (0%)	1 (2.4%)	25 (62.5%)	
Zinc (µmol/L)	11.9 ± 1.8	10.1 ± 2.2	8.2 ± 1.8	8.2 ± 2.3	0.000
WBC (g/L)	5.83 ± 2.36	5.68 ± 2.04	7.27 ± 3.50	14.86 ± 10.67	0.000
CRP (mg/L)	1.6 ± 0.9	13.6 ± 25.0	87.3 ± 82.2	125.5 ± 100.8	0.000
PCT (ng/mL)	0.03 ± 0.07	0.04 ± 0.05	1.30 ± 4.67	2.88 ± 7.01	0.004
LDH (U/L)	180.4 ± 37.6	203.1 ± 66.2	361.8 ± 159.4	512.2 ± 419.3	0.000
Ferritin (ng/L)	61.0 ± 61.2	127.0 ± 240.2	854.4 ± 1373.5	1323.8 ± 2029.9	0.000

Data are presented as mean ± SD. Comparisons among the groups were performed using the one-way analysis of variance (ANOVA). Nominal data were analyzed by chi-square test. A *p* value of <0.05 was considered statistically significant.

**Table 3:** Demographic and laboratory parameters of hospitalized COVID-19 patients according to the survival status.

Parameters	Hospital Discharge	Death	*p*
Age (years) (*n*)	63.7 ± 14.3 (55)	74.2 ± 14.5 (26)	0.003
Sex (M/F)	23/32	16/10	0.253
ICU required (%)	15 (37.5%)	25 (62.5%)	
Zinc (µmol/L)	8.4 ± 1.8	7.9 ± 2.6	0.317
WBC (g/L)	7.77 ± 3.59	17.88 ± 11.98	0.000
CRP (mg/L)	82.6 ± 76.9	155.4 ± 107.7	0.004
PCT (ng/mL)	1.09 ± 4.12	4.10 ± 8.33	0.032
LDH (U/L)	347.3 ± 136.9	623.8 ± 488.7	0.000
Ferritin (ng/L)	966.2 ± 1939.2	1340.1 ± 1184.6	0.368

Data are presented as mean ± SD. The statistical analysis of differences between two groups was performed using two-tailed Student’s *t*-test with a confidence level of 95%. A *p* value of <0.05 was considered statistically significant.

## Data Availability

The data presented in this study are available on request from the corresponding author.
